# A Novel Approach Using Microarray Testing as a Screening Method with Clinical Validation Using Whole-Genome Sequencing and Karyotyping for Identifying 46,XX Testicular Differences of Sex Development

**DOI:** 10.3390/diagnostics16050706

**Published:** 2026-02-27

**Authors:** Takeshi Ozeki, Yuka Nakano, Ayumu Ishigaki, Yoichi Kawashima, Charles W. Crawford, David D. Ordinario, Iri Sato-Baran, Toshihiko Higashida

**Affiliations:** 1 Genesis Institute of Genetic Research, Genesis Healthcare Co., Yebisu Garden Place Tower 26F, 4-20-3 Ebisu, Shibuya-ku, Tokyo 150-6026, Japan; takeshi_ozeki@genesis-healthcare.jp (T.O.); yuka_nakano@genesis-healthcare.jp (Y.N.); ayumu_ishigaki@genesis-healthcare.jp (A.I.); yoichi_kawashima@genesis-healthcare.jp (Y.K.); charles_crawford@genesis-healthcare.jp (C.W.C.); david_ordinario@genesis-healthcare.jp (D.D.O.); sato@genesis-healthcare.jp (I.S.-B.); 2 Souseikai Medical Group, Medical Clinic Ebisu, Yebisu Garden Place Tower 26F, 4-20-3 Ebisu, Shibuya-ku, Tokyo 150-6026, Japan

**Keywords:** 46,X,der(X)t(X;Y)(p22.3;p11.1), chromosomal translocation, *SRY*, XX male, direct-to-consumer genetic testing service

## Abstract

**Background:** Microarray testing is commonly used as a screening method for phenotypic traits and common diseases and for genome-wide association studies (GWASs). Despite the known limitations, microarray services can potentially be used as a prescreening tool for chromosomal disorders, which affect approximately 0.4–0.6% of the world population, followed by further clinical diagnostic methods when appropriate. **Case Presentation**: Here we present a case study of a male subject in his 40s who underwent direct-to-consumer (DTC) genetic testing that utilized microarray, which revealed the absence of Y chromosome haplogroup data despite possessing a typical male phenotype. Subsequent medical consultation, whole-genome sequencing (WGS), and chromosomal analysis confirmed a diagnosis of 46,XX testicular differences of sex development (DSD, formerly XX male syndrome) characterized by the presence of Y chromosome-derived genomic material, including the *SRY* gene. An initial microarray test gave an indeterminate result for the Y chromosome call rate and an X chromosome heterozygosity result that aligned with the female average. These indeterminate results, coupled with the subject’s male phenotype, led to further testing—WGS, karyotyping, fluorescence in situ hybridization using an *SRY* Probe, and endocrine testing. From these results, the subject was diagnosed with 46,XX testicular DSD. **Conclusions**: To our knowledge, this represents the first reported case where 46,XX testicular DSD was diagnosed starting from a DTC test which led to medical consultation and comprehensive genomic and cytogenetic analysis. This case underscores the potential diagnostic value of consumer-initiated DTC microarray screening in the era of genomic medicine and for supporting social needs such as gender confirmation for sports.

## 1. Introduction

Chromosomal disorders affect approximately 0.4–0.6% of the world population, and over half of all disorders are thought to go undetected [1,2]. These unidentified genetic disorders include those that do not manifest with a clearly visible phenotype, as well as adult-onset diseases, and often fail to lead to appropriate testing and treatment. However, the rise of personalized healthcare and genomic medicine has led to an increase in testing driven by the demand for personalized genetic information [3,4,5,6,7,8]. Concurrently, increased awareness of the role of genetics in personal health and steadily decreasing testing costs have facilitated a rise in direct-to-consumer (DTC) microarray genetic testing services [9,10,11]. Although these tests offer increased accessibility and affordability, there are concerns stemming from the variability between testing labs and the lack of reliable professional medical advice to verify results and provide assistance in interpretation [12,13,14]. Furthermore, while microarray testing has been used for genetic screening and genome-wide association studies (GWASs) for identifying genetic risk factors for many complex diseases and traits, it is not solely used for clinical diagnostics [15,16,17,18]. Despite their known limitations, DTC microarray services can potentially be used as a prescreening tool to find chromosomal disorders that would have otherwise gone undetected when combined with professional medical oversight and reliable, standardized testing protocols.

One example of a chromosomal disorder that can be screened for by DTC genetic testing is 46,XX testicular differences of sex development (DSD, also known as disorders of sex development, formerly XX male syndrome) [19,20,21], also known as de la Chapelle syndrome, occurring in approximately 4 out of every 100,000 male newborns [22,23]. There is no strong evidence to suggest a significantly higher or lower incidence rate in Japan compared to other ethnic populations. There is currently no robust epidemiological data indicating unique genetic predispositions or a distinct pattern of 46,XX testicular DSD within the Japanese population that significantly differs from global observations. The fundamental genetic mechanisms and clinical presentations appear consistent across ethnic groups. Normally, the male phenotype is determined by the *SRY* (Sex-determining Region Y) gene, located on the short arm of the Y chromosome [24]. However, in individuals with 46,XX testicular DSD, the *SRY* gene is often translocated onto an X chromosome during meiosis [25,26]. However, because ~90% of affected individuals present a normal male phenotype, the number of reported cases is small, and many aspects of the clinical presentation remain unclear [27]. With DTC microarray testing, previously undetected cases of 46,XX testicular DSD may be uncovered due to increased access by individuals.

Here, we present a case study where the results of a DTC genetic test taken by a male subject in his 40s indicated the absence of a Y chromosome. Under medical supervision, whole-genome sequencing (WGS) was conducted, confirming translocation of a Y chromosome short arm containing the SRY gene to the terminal region of an X chromosome. By combining conventional karyotyping with microarray analysis (widely used in DTC genetic testing) and WGS (commonly used in clinical diagnostics), a comprehensive view of the individual’s genome was constructed, and a definitive diagnosis was given. This case illustrates how consumer-initiated genetic testing can contribute to the diagnosis of unrecognized chromosomal abnormalities, underscoring the evolving role of genomic medicine in everyday healthcare.

## 2. Case Presentation

### 2.1. Subject Profile

The subject of this study was a 41-year-old phenotypic male with no notable developmental abnormalities experienced during childhood and no physical or psychological issues experienced as an adult. The subject’s father was frail from birth and died of testicular choriocarcinoma at the age of 29. The subject’s mother is 72 years old (at the time of writing) and is alive and well. The subject has no siblings. The subject married at the age of 29, has had no issues with sexual function, and has no children. According to the subject, he has led a normal life, has been employed full-time at a game development company, has reported no awareness of any physical abnormalities, and has had no notable history of illness.

At the time of writing, the subject was 166 cm (approximately at the 18th percentile of the Japanese population), 52.5 kg (BMI 19.1). On visual inspection, the subject exhibited a typical male body habitus and secondary sexual characteristics, including facial hair, with no outward abnormalities. As the visit was for the purpose of testing and explanation of test results during a clinical consultation, no additional physical examination findings were recorded.

### 2.2. Discovery of Genetic Abnormality

Genesis Healthcare Co. (Tokyo, Japan) (hereinafter referred to as “GH”) provides direct-to-consumer (DTC) genetic testing services based on microarrays. Among these, GeneLife Haplo3.0 Ancestry DNA Kit reports on ethnic composition percentages and the migration history of ancestors through MT and Y-haplogroup analysis. The subject initially purchased the GeneLife Haplo3.0 Ancestry DNA Kit from GH. Following the provided instructions, the subject collected a saliva sample at home and submitted it to GH’s laboratory for analysis. After receiving the test results, the subject contacted GH to inquire why no paternal (Y chromosome) haplogroup was detected despite being male. After confirming that no human error had occurred, the subject’s test results were re-examined, revealing a discrepancy: while the subject was phenotypically male, the genetic data suggested a female karyotype.

### 2.3. Microarray Analysis

Initially, a detailed examination of sex chromosomes was performed through microarray testing. DNA was extracted from saliva and purified with the MGIEasy Nucleic Acid Extraction Kit (MGI Tech Co., Ltd., Shenzhen, China). Microarray analysis was performed using an Infinium CoreExome-24 v1.4 kit (Illumina, San Diego, CA, USA) and iScan System (Illumina, San Diego, CA, USA).

The call rate for a Y chromosome was measured to be ~39% (1729 out of 4419 SNPs), which is lower than the average for males (~71%; *n* = 27,181; 3177/4419 SNPs) but higher than the average for females (~26%; *n* = 40,509; 1167/4419 SNPs) (Table 1). Heterozygosity on an X chromosome was ~16% (2417 out of 14,924 SNPs), which is higher than the male average (~1%; *n* = 27,181; 134/14,924 SNPs) and closely aligned with the female average (~16%; *n* = 40,509; 2417/14,924 SNPs) (Table 1). It is noted that the array covers the pseudo autosomal region (PAR), shared by the X and the Y chromosome and amounting to 16.1% of the “X chromosome” SNP calls. In addition, this subject possessed the Y chromosome regions from 0.18 to 9.31 Mb, which cover the *SRY* gene.

### 2.4. Whole-Genome Sequencing (WGS) Analysis

Subsequently, WGS was performed to further analyze the sex chromosomes. DNA was extracted from saliva and purified with Oragene prepIT (DNA Genotek, Ottawa, ON, Canada). Libraries were prepared using the MGIEasy FS DNA Library Prep Set (MGI Tech Co., Ltd., Shenzhen, China). WGS analysis was performed using a DNBSEQ-T7 (MGI Tech Co., Ltd., Shenzhen, China) for sequencing and the Illumina DRAGEN DNA pipeline (Illumina, San Diego, CA, USA) for read mapping. The mapping status of the subject’s sex chromosomes was examined in detail with the Integrative Genomics Viewer, Version 2.17 (UC San Diego, San Diego, CA, USA; Broad Institute, Cambridge, MA, USA) [28,29,30,31].

**Figure 1:**
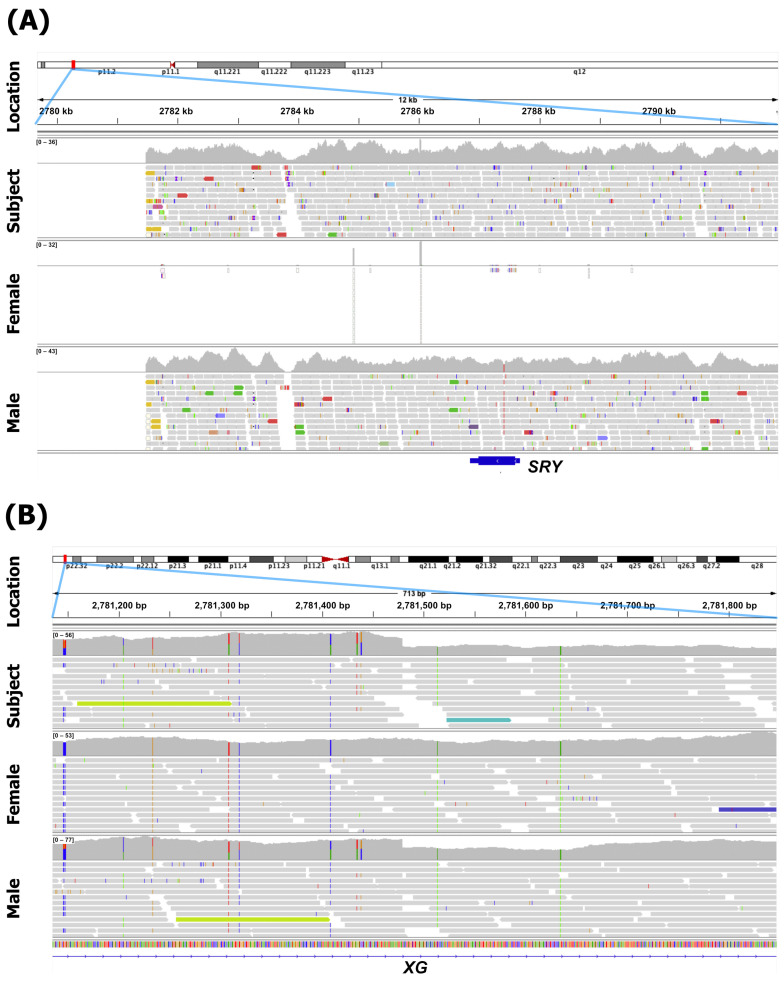
Comparison of the subject’s sex chromosomes with typical male XY and typical female XX chromosomes. (**A**) The mapping status of the region immediately following PAR1 on the Y chromosome was shown using the Integrative Genomics Viewer. In the reference sequence, the PAR1 region (1–2781.5 kb) is masked on the Y chromosome, so it is not mapped in males or females. The region immediately following PAR1 in the subject contained the portion of the Y chromosome including the *SRY* gene (blue bar), as seen in the male. (**B**) The mapping status immediately after the PAR1 region of the X chromosome was shown. In the PAR1 region (1–2781.5 kb) of the X chromosome, all exhibit heterozygosity. Immediately after the PAR1 region of the subject, about 50% reduction in coverage and disappearance of heterozygosity were observed, as in the male, indicating that the subject had one normal X chromosome.

**Figure 2:**
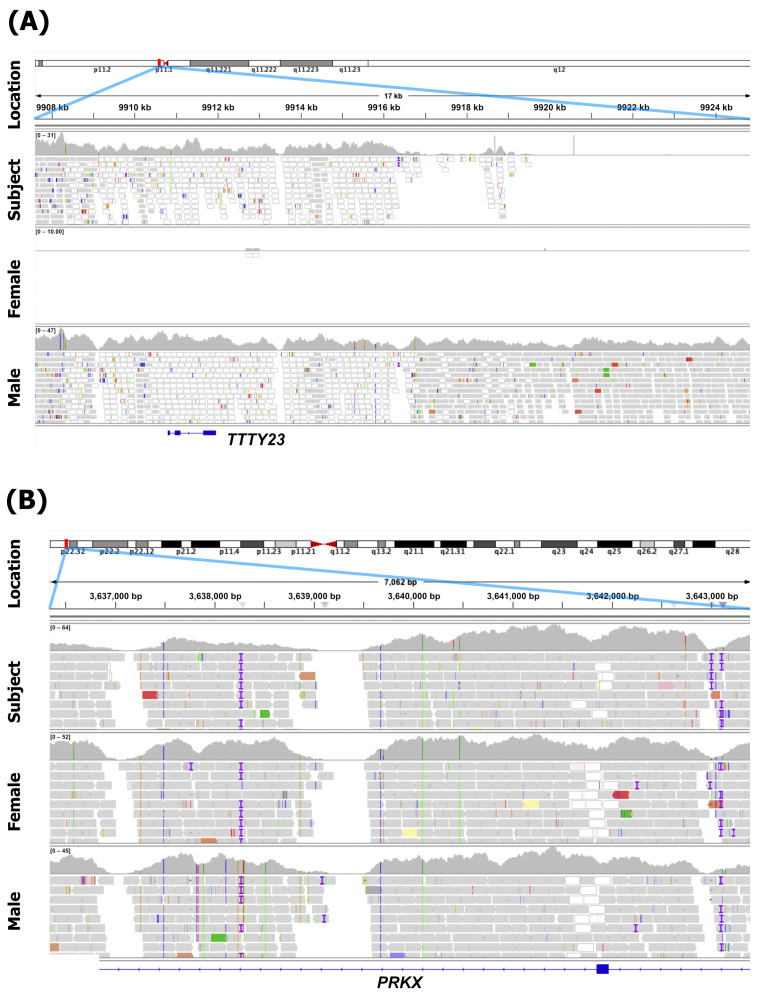
Comparison of the subject’s sex chromosomes with typical male XY and typical female XX chromosomes. (**A**) The mapping status of the area surrounding the Y chromosome centromere (p11.1). For the subject, the region beyond the short arm terminal of the Y chromosome was not mapped, and the long arm appeared to be missing. (**B**) The mapping status of the p22.32 region of the X chromosome. The region beyond p22.32 in the subject was similar to that in females, with increased coverage and heterozygosity.

A portion of the Y chromosome normally on the Y chromosome’s short arm, including the *SRY* gene, was successfully mapped immediately downstream of the PAR1 region (Figure 1A). On an X chromosome, a pattern consistent with the presence of a single normal X chromosome was observed: immediately downstream of the PAR1 region, read coverage was reduced by half and heterozygosity was absent, similar to male control samples (Figure 1B). However, beyond the distal region of a Y short arm (corresponding to Yp11.1), sequences appeared to be absent (Figure 2A). In contrast, from approximately position p22.32 on an X chromosome, read depth doubled, matching the profile seen in a female control (Subject A), indicating the presence of two copies of an X chromosome from that point onward (Figure 2B). As the PAR1 region is located near Xp22.33 on an X chromosome [32], the deletion on one of the X chromosomes was presumed to be a microdeletion limited to the region between the 5′ terminus and p22.32. Thus, the WGS result in microarray format was written according to the standardized international nomenclature of ISCN 2024 as seq[GRCh38] ins(X;Y)(p22.32;p11.31) (chrX:g.2781480_3639500ins [chrY:g.2781500_9016500]).

### 2.5. G-Banding, Fluorescence In Situ Hybridization (FISH), and Endocrine Testing

**Figure 3:**
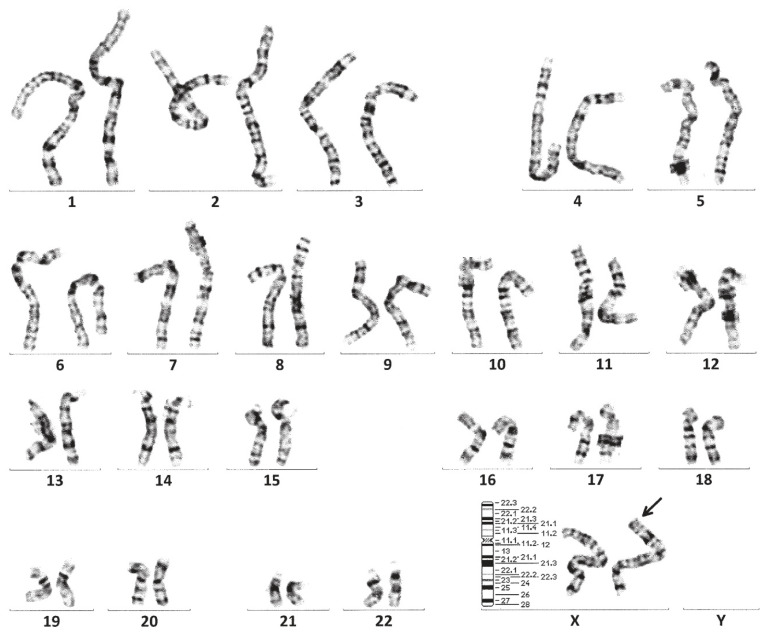
G-banding results for subject. No Y chromosome was detected in any of the metaphase spreads examined, while two X chromosomes were observed. One of the X chromosomes exhibited an abnormal staining band (by arrow) on the short arm (Xp), which is not typically present. No other chromosomal abnormalities were identified.

**Figure 4:**
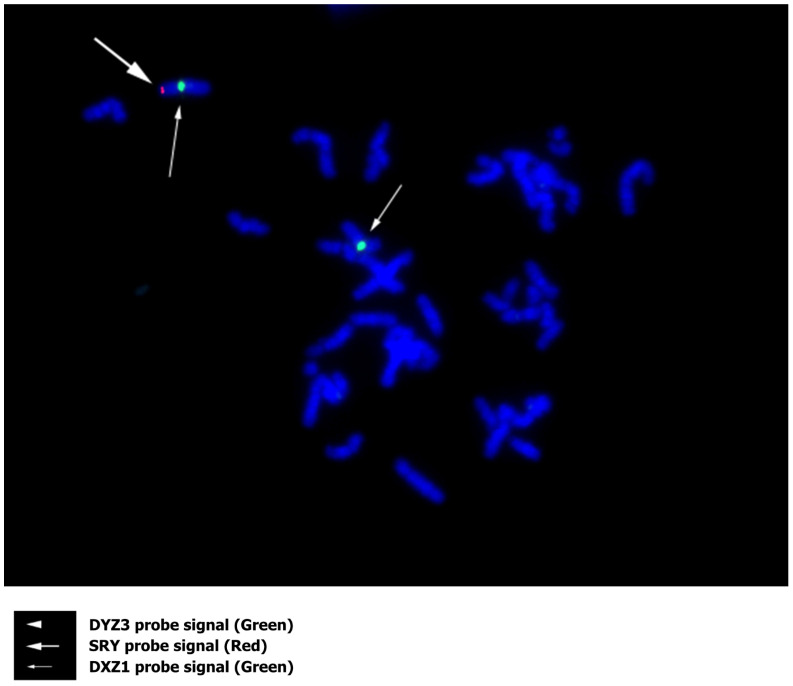
Fluorescence in situ hybridization (FISH) for the subject. One chromosome showed positive DXZ1 (white thin arrow and green signal) and positive *SRY* (white thick arrow and red signal) signals, while another chromosome with positive DXZ1 signal lacked any *SRY* signal. DYZ3 (white arrowhead and green signal) was not detected.

To confirm the findings of the WGS analysis, karyotyping though G-banding (Figure 3) and fluorescence in situ hybridization (FISH) using an *SRY* probe (Figure 4) were performed. G-banding, FISH, and endocrine testing were all performed by an accredited external medical testing laboratory (LSI Medience, Tokyo, Japan). Human blood samples were collected via venipuncture using TERUMO Venoject II EDTA Vacutainer tubes (TERUMO, Tokyo, Japan). Three 5 mL vials were drawn from one person to obtain a sufficient concentration of chromosomal DNA. Immediately after each collection, all blood samples were placed in styrofoam containers with icepacks to maintain a temperature below 22 °C during transport to the external medical testing laboratory. After completion, test results and analysis were received electronically.

No Y chromosome was detected in any of the metaphase spreads examined while two X chromosomes were observed. One of the X chromosomes exhibited an abnormal staining band on the short arm (Xp), which is not typically present. At this site, a positive signal for the *SRY* probe was detected. In addition to the *SRY* probe, DYZ3 and DXZ1 probes were used. Two DXZ1 signals were detected, further confirming the presence of two X chromosomes, while no DYZ3 signal was detected. No other chromosomal abnormalities were identified. Thus, the WGS result related to translocation, karyotype, and FISH was written according to the standardized international nomenclature of ISCN 2024 as 46,XX.seq der(X)ins(X;Y)(p22.32;p11.31)(SRY+).

Endocrine testing was performed to provide further confirmation of the subject’s condition. Levels of free testosterone, total testosterone, dehydroepiandrosterone sulfate (DHEA-S), and growth hormone (GH) all fell within reference ranges (Table 2). However, luteinizing hormone (LH) and follicle-stimulating hormone (FSH) levels were significantly higher than normal, with LH at 21.61 mIU/mL (reference upper limit: 5.72 mIU/mL) and FSH at 51.19 mIU/mL (reference upper limit: 8.30 mIU/mL).

## 3. Discussion

In microarray testing, although these results indicated that the subject was genetically female, the subject was phenotypically male. Thus, the evidence suggested the presence of two X chromosomes and partial loss of the Y chromosome or the possibility that the SRY gene had been translocated to another chromosome. The deduced karyotype including the microarray data was arr[GRCh37] Xp22.33p22.3(181,779_3,555,302) × 1, Yp11.32p11.1(181,779_9,314,387) × 1 (Table 1).

WGS suggests that the SRY-containing region of a Y chromosome short arm was translocated to the 5′ end of one X chromosome, while the other X chromosome remained structurally normal. Results of G-banding and FISH suggested that the subject carried one normal X chromosome and one structurally altered X chromosome that includes a translocated segment from the short arm of the Y chromosome containing the *SRY* locus. Short stature can be observed in 46,XX testicular DSD due to a deficiency of the *GCY* gene, which is located on the long arm of the Y chromosome [33]. Although the subject’s height of 166 cm is similar to that reported in individuals with XX male syndrome, a definitive causal link cannot be determined. Additionally, deletions within the AZFa, AZFb, or AZFc regions, all crucial for spermatogenesis, are suggested to lead to azoospermia or oligospermia (though these tests were not performed in this subject).

In endocrine testing, compared to the reference range for Japanese adult males in their 40s (7.7–21.6 pg/mL), LH and FSH levels were elevated, and free testosterone was relatively low [34]. Elevated LH and FSH levels are frequently reported in 46,XX testicular DSD individuals, often accompanied by reduced testosterone [35,36,37]. The negative feedback regulation of LH and FSH is thought to involve testosterone secreted by Leydig cells and inhibin secreted by Sertoli cells [38]. The relatively low testosterone levels in this subject suggest a dysfunction in Leydig and/or Sertoli cell function, resulting in insufficient feedback suppression of LH and FSH. While the direct cause of low testosterone in 46,XX testicular DSD individuals remains unclear, similar hormonal patterns are observed in individuals with a 47,XXY karyotype (Klinefelter syndrome) [39], suggesting a contribution from the presence of additional X chromosomes.

DTC microarray services hold potential as a preliminary screening tool for detecting chromosomal abnormalities that would otherwise remain undetected. However, a limitation is that standard DTC genetic testing does not incorporate algorithms designed to detect chromosomal abnormalities not included in the standard testing panel. Consequently, inconsistencies in test results like those observed in this case—where a male subject failed to yield Y-haplogroup analysis results—risk being overlooked.

## 4. Conclusions

As microarray genetic testing becomes more affordable and widespread, its utility as a prescreening tool continues to grow. In addition to guiding individuals toward appropriate diagnostic services and treatments, microarray testing may also help uncover the prevalence of rare conditions with subtle or atypical phenotypes. This case illustrates how DTC microarray testing can identify chromosomal abnormalities such as 46,XX testicular DSD, which often go undiagnosed and are not routinely screened for. Importantly, the microarray and WGS results were consistent with karyotype analyses (G-banding and FISH), supporting the reliability of high-quality data generated by private testing laboratories. With appropriate quality control and interpretive frameworks, DTC-derived genetic data can serve as a valuable resource for both clinical and research applications, conducted under subject request and informed consent. When adjusted for potential sampling biases, aggregated DTC datasets could enhance our understanding of the prevalence and distribution of rare genetic disorders, providing benefits beyond the individuals who purchase the tests.

In conclusion, we have demonstrated how consumer-initiated DTC microarray genetic testing utilized as a screening tool contributed to the diagnosis of an unrecognized chromosomal abnormality. This case underscores the evolving role of genomic medicine not only for the detection of chromosomal disorders but also for everyday healthcare. In the future, DTC testing may serve as an additional platform for early screening of chromosomal and genetic abnormalities, offering clinicians more timely access to valuable information.

## Figures and Tables

**Table 1:** Characteristics of the subject’s sex chromosomes with typical male XY and typical female XX chromosomes based on microarray testing.

Entry	Male (Mean ± SD) (*n* = 27,181)	Female (Mean ± SD) (*n* = 40,509)	Subject
Y chromosome call rate ^1^	71.9 ± 1.2%	26.4 ± 2.5%	39.1%
X chromosome heterozygosity ^2^	0.9 ± 0.1%	16.2 ± 0.8%	16.2%
Deduced sex	Male	Female	Female?
Result in accordance with ISCN 2024	arr[GRCh37] (X,Y)×1,(1–2)×2	arr[GRCh37] (X,1–22) × 2	arr[GRCh37] Xp22.33p22.3(181,779_3,555,302) × 1, Yp11.32p11.1(181,779_9,314,387) × 1

^1^ The Y chromosome call rate was calculated with the following equation: $\frac{\#\;of\;variants\;(Y)}{4419\;tested\;variants\;(Y)}*100\;(\%)$. ^2^ The X chromosome heterozygosity was calculated with the following equation: $\frac{\#\;of\;heterozygote\;variants\;(X)}{14,924\;tested\;variants\;(X)}*100\;(\%)$.

**Table 2:** Endocrine test results.

Test Item ^1^	Result (Unit)	Reference Value (Unit)
Free testosterone	5.6 pg/mL	4.7–21.6 pg/mL
Testosterone	4.76 ng/mL	1.92–8.84 ng/mL
DHEA-S	201 μg/dL	123–422 μg/dL
GH	0.78 ng/mL	<2.47 ng/mL
LH	21.61 mIU/mL	0.79–5.72 mIU/mL
FSH	51.19 mIU/mL	2.00–8.30 mIU/mL

^1^ DHEA-S, dehydroepiandrosterone sulfate; GH, growth hormone; LH, luteinizing hormone; FSH, follicle-stimulating hormone.

## Data Availability

The data presented in this study are available in SRA at https://www.ncbi.nlm.nih.gov/bioproject/PRJNA1300322 (accessed on 29 December 2025), reference number PRJNA1300322.

## Abbreviations

The following abbreviations are used in this manuscript:

GWAS	Genome-wide association study
DTC	Direct to consumer
DSD	Differences of sex development
WGS	Whole-genome sequencing
SRY	Sex-determining Region Y
GH	Genesis Healthcare Co.
SNP	Single-nucleotide polymorphism
PAR	Pseudo autosomal region
FISH	Fluorescence in situ hybridization
DHEA-S	Dehydroepiandrosterone sulfate
GH	Growth hormone
LH	Luteinizing hormone
FSH	Follicle-stimulating hormone
